# A structural analysis of the regulatory domain from the cGMP-dependent protein kinase Iα

**DOI:** 10.1186/1471-2210-11-S1-P53

**Published:** 2011-08-01

**Authors:** Brent W Osborne, Andrew T Menke, Donald K Blumenthal, Wolfgang R Dostmann

**Affiliations:** 1 Department of Pharmacology, College of Medicine, University of Vermont, Burlington, VT 05405, USA; 2 Department of Pharmacology & Toxicology, University of Utah, Salt Lake City, Utah 84112, USA

## Background

The cGMP-dependent protein kinase (PKG) has two tandem cyclic nucleotide binding (CNB) domains which act as the primary intracellular receptor for cGMP [1,2]. PKG exhibits a homodimeric rod-like structure which undergoes significant molecular rearrangements upon the binding of cGMP [3-5]. However, a detailed structural analysis of the core regulatory elements inherent to PKG is still required.

## Results

**Figure 1:**
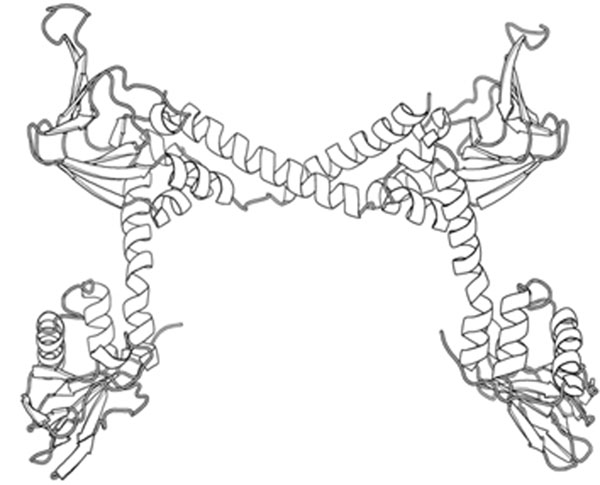
Overall fold of PKG^78-355^. The crystal structure of the PKG regulatory domain identifies a novel allosteric interface between PKG^78-355^ protomers.

We recently solved a crystal structure of the two cGMP binding sites from PKG Iα in order to highlight the atomic details of the regulatory domain. This PKG^78-355^ structure is free of cGMP and presents the protein in an elongated conformation. A surprising dimeric arrangement between PKG^78-355^ protomers is orchestrated via hydrophobic contacts between a novel helical element C-terminal to the second cGMP binding site (the switch helix) and the opposite CNB domain B (Figure 1). Small angle X-ray scattering (SAXS) of PKG^78-355^ suggests an overall molecular dimension of ~130 Å, consistent with the maximal linear dimension observed in our crystal structure. Upon incubation with cGMP, PKG^78-355^ contracted to ~95 Å. This molecular compaction was not observed in a construct lacking the switch helix (PKG^78-326^), suggesting the additional importance of the switch helix in mediating cGMP-specific conformational changes inherent to the regulatory domain.

## Conclusion

Overall, these studies provide the first atomic resolution model of tandem cGMP binding domains and expand our understanding of the allosteric mechanisms surrounding PKG activation.
